# Systemic Regulation of RAS/MAPK Signaling by the Serotonin Metabolite 5-HIAA

**DOI:** 10.1371/journal.pgen.1005236

**Published:** 2015-05-15

**Authors:** Tobias Schmid, L. Basten Snoek, Erika Fröhli, M. Leontien van der Bent, Jan Kammenga, Alex Hajnal

**Affiliations:** 1 University of Zurich, Institute of Molecular Life Sciences, Zurich, Switzerland; 2 PhD Program in Molecular Life Sciences, University and ETH Zurich, Zurich, Switzerland; 3 Laboratory of Nematology, Wageningen University, Wageningen, The Netherlands; Stanford University Medical Center, UNITED STATES

## Abstract

Human cancer is caused by the interplay of mutations in oncogenes and tumor suppressor genes and inherited variations in cancer susceptibility genes. While many of the tumor initiating mutations are well characterized, the effect of genetic background variation on disease onset and progression is less understood. We have used *C*. *elegans* genetics to identify genetic modifiers of the oncogenic RAS/MAPK signaling pathway. Quantitative trait locus analysis of two highly diverged *C*. *elegans* isolates combined with allele swapping experiments identified the polymorphic monoamine oxidase A (MAOA) gene *amx-2* as a negative regulator of RAS/MAPK signaling. We further show that the serotonin metabolite 5-hydroxyindoleacetic acid (5-HIAA), which is a product of MAOA catalysis, systemically inhibits RAS/MAPK signaling in different organs of *C*. *elegans*. Thus, MAOA activity sets a global threshold for MAPK activation by controlling 5-HIAA levels. To our knowledge, 5-HIAA is the first endogenous small molecule that acts as a systemic inhibitor of RAS/MAPK signaling.

## Author Summary

Mutations that activate a RAS oncogene are found in a large proportion of human cancers. In this study, we have used the roundworm *Caenorhabditis elegans* (*C*. *elegans*) as a model to investigate how the genetic composition of the animal affects the outcome of oncogenic RAS mutations that activate the MAPK pathway. By comparing the effects of activated RAS/MAPK signaling in two genetically different *C*. *elegans* strains, we have identified the monoamine oxidase A (MAOA) gene *amx-2* as a negative regulator of RAS/MAPK signaling. MAOA enzymes are primarily known to catalyze the degradation of the neurotransmitters dopamine and serotonin. Here, we show that a specific serotonin degradation product that is produced by MAOA (5-HIAA) inhibits RAS signaling in different organs of *C*. *elegans*. Thus, by producing the inhibitory serotonin metabolite 5-HIAA the MAOA enzyme systemically controls the activation of the RAS/MAPK pathway.

## Introduction

Human cancer is a complex polygenic disease caused by somatic mutations in oncogenes and tumor suppressor genes together with inherited polymorphisms in cancer susceptibility genes. Many of the oncogenes and tumor suppressor genes that are mutated in different cancer types have been investigated in detail. However, relatively little is known about the effect of the genetic background on disease onset and progression. It thus remains a challenge to identify functional links between oncogenic traits and associated natural variants [1,2].

The components of the RAS/MAPK signaling pathway are mutated in a large fraction of human tumors. In particular, activating (“gain-of-function”) mutations in HRAS and KRAS are among the most prevalent tumor initiating mutations found in human cancer cells [3]. Thanks to the strong conservation of this pathway in metazoans, genetic studies in model organisms, such as the nematode *Caenorhabditis elegans*, have provided important insights into various factors modulating RAS/MAPK signaling [4]. Moreover, *C*. *elegans* has become a platform species for quantitative genetic analyses of various phenotypes and pathways in order to identify and characterize polymorphic genes [5,6]

In this study, we have used quantitative genetics to explore how the genetic background affects the phenotypes caused by the activating G13E (*n1046*) mutation in the *C*. *elegans ras* gene *let-60* [7]. The *n1046* mutation is homologous to the HRAS and KRAS mutations that are frequently found in human cancer cells [3]. For the purpose of this study, we compared RAS/MAPK signaling in two highly diverse genetic backgrounds, *C*. *elegans* varieties Bristol (N2) and Hawaii (CB4856) [8]. Compared to the reference strain N2, the Hawaiian CB4856 strain on average contains one polymorphism every 412 bp with around 75% of all genes carrying at least one coding polymorphism [9].

**Fig 1:**
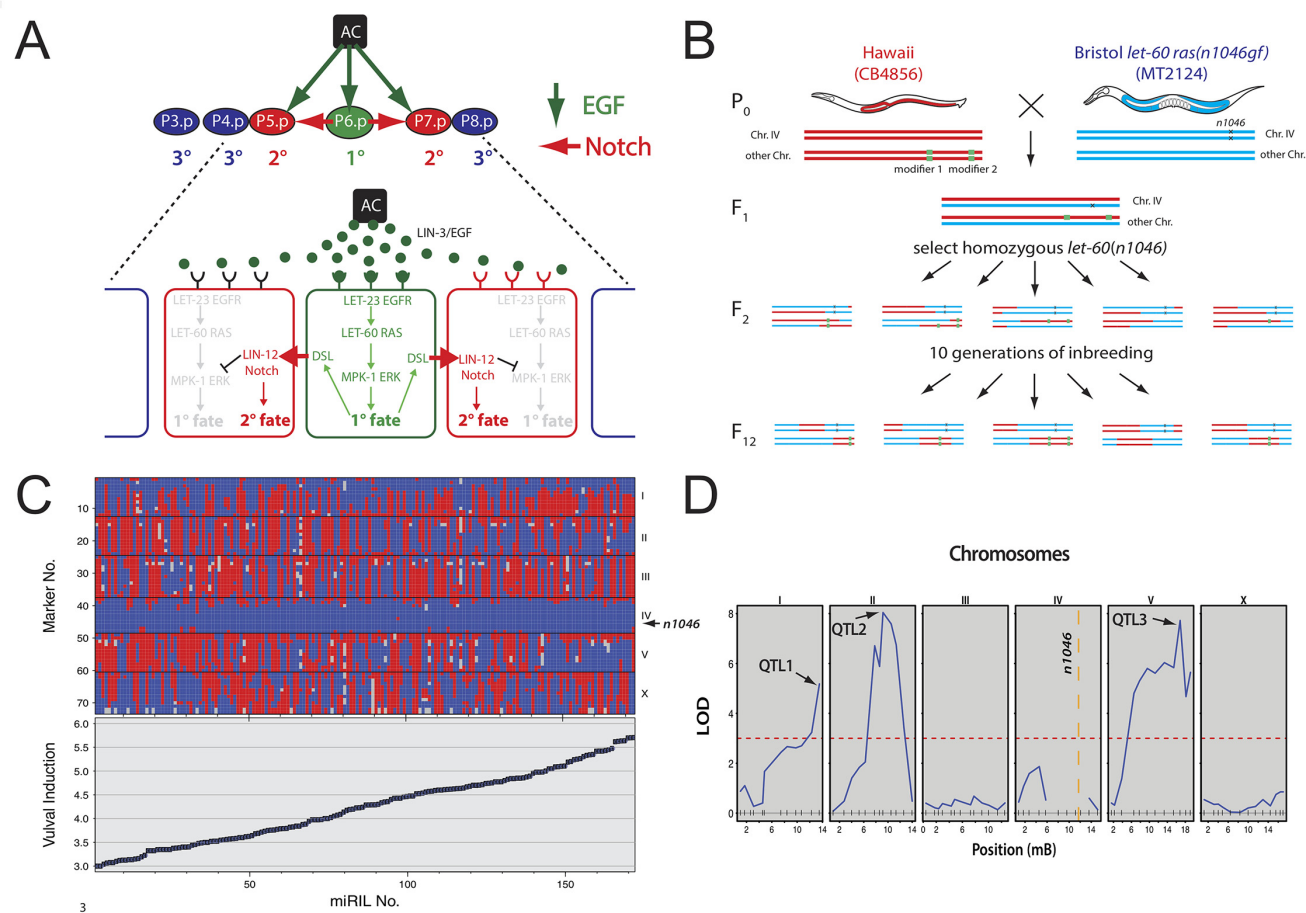
QTL mapping of *let-60 ras* modifiers. **(A)** RAS/MAPK signaling induces three VPCs. P6.p receives most of the inductive EGF signal from the anchor cell and activates the EGFR/RAS/MAPK pathway inducing the 1° cell fate (green arrows). Lateral signaling via the Notch pathway induces the 2° cell fate in the neighboring VPCs P5.p and P7.p (red arrows). The remaining VPCs (blue) adopt the non-vulval 3° cell fate. **(B)** Crossing scheme to generate the *let-60(n1046gf)* miRILs. Hawaii males (red) were crossed with Bristol *let-60(n1046gf)* mutants (blue). For each example animal, the two chromosomes IV carrying the *n1046* mutation and another arbitrary chromosome pair are shown. Random segregation of the two parental genomes was allowed except for the *let-60(gf)* mutation that was kept homozygous from F2 generation onwards. After ten generations of self-fertilization to drive all regions to homozygosity, 228 independent miRILs were obtained. **(C)** Genotypes and phenotypes of the *let-60(gf)* miRILs sorted by increasing VI. Genotypes determined by FLP mapping [15] are plotted on the y-axis versus the miRIL numbers on the x-axis. Hawaii genotypes are indicated with red, Bristol genotypes with blue and missing genotypes with gray colors. The VIs for each miRIL are shown below the genotypes. Error bars indicate the standard error of the mean. **(D)** QTL mapping identified three regions (QTL1 through QTL3) above the threshold LOD score of 3 (dotted red line). In each of the panels showing chromosomes I through X, the locations of the FLP markers used for genotyping are indicated on the x-axis with vertical lines. For the exact locations of the FLPs used, see Materials and Methods and [15].

To measure the activity of the RAS/MAPK pathway in different genetic backgrounds, vulval induction can be used as a quantifiable and reproducible readout. During vulval development, the anchor cell in the somatic gonad secretes the EGF-like ligand that activates via an EGFR family receptor tyrosine kinase the RAS/MAPK signaling pathway in the adjacent vulval precursor cells (VPCs) [10]. In combination with a lateral NOTCH signal, RAS/MAPK signaling induces three of the six VPCs to adopt a 2°-1°-2° pattern of vulval cell fates (**Fig 1A**). Mutations that hyperactivate RAS/MAPK signaling, such as the *n1046* allele, cause the differentiation of more than three and up to six VPCs and a Multivulva phenotype, while mutations that reduce RAS/MAPK signaling result in the induction of fewer than three VPCs and a Vulvaless phenotype. Hence, the average number of induced VPCs per animal, the vulval induction (VI), is a quantitative measure of RAS/MAPK signaling output in the VPCs [10,11]. Besides the vulva, RAS/MAPK signaling is activated in a variety of other tissues in *C*. *elegans* at different developmental stages, such as the meiotic germ cells in the hermaphrodite gonads, the excretory duct cell precursor in the embryo or the chemosensory neurons during olfaction in adults [4]. Using a quantitative genetics approach, we aimed at identifying globally acting as well tissue-specific modifiers of RAS/MAPK signaling. Here, we describe the identification of the polymorphic monoamine oxidase *amx-2* gene as a global negative regulator of the RAS/MAPK pathway. *amx-2* encodes a mitochondrial monoamine oxidase type A (MAOA) that catalyzes the oxidative deamination of biogenic amines such as dopamine (DA) and serotonin (5-HT) [12]. We further show that AMX-2 activity in intestinal cells controls the levels of the serotonin metabolite 5-hydroxyindoleacetic acid (5-HIAA), which acts as systemic inhibitor of MAPK phosphorylation.

## Results

### Identification of RAS/MAPK modifiers by quantitative *C*. *elegans* genetics

**Fig 2:**
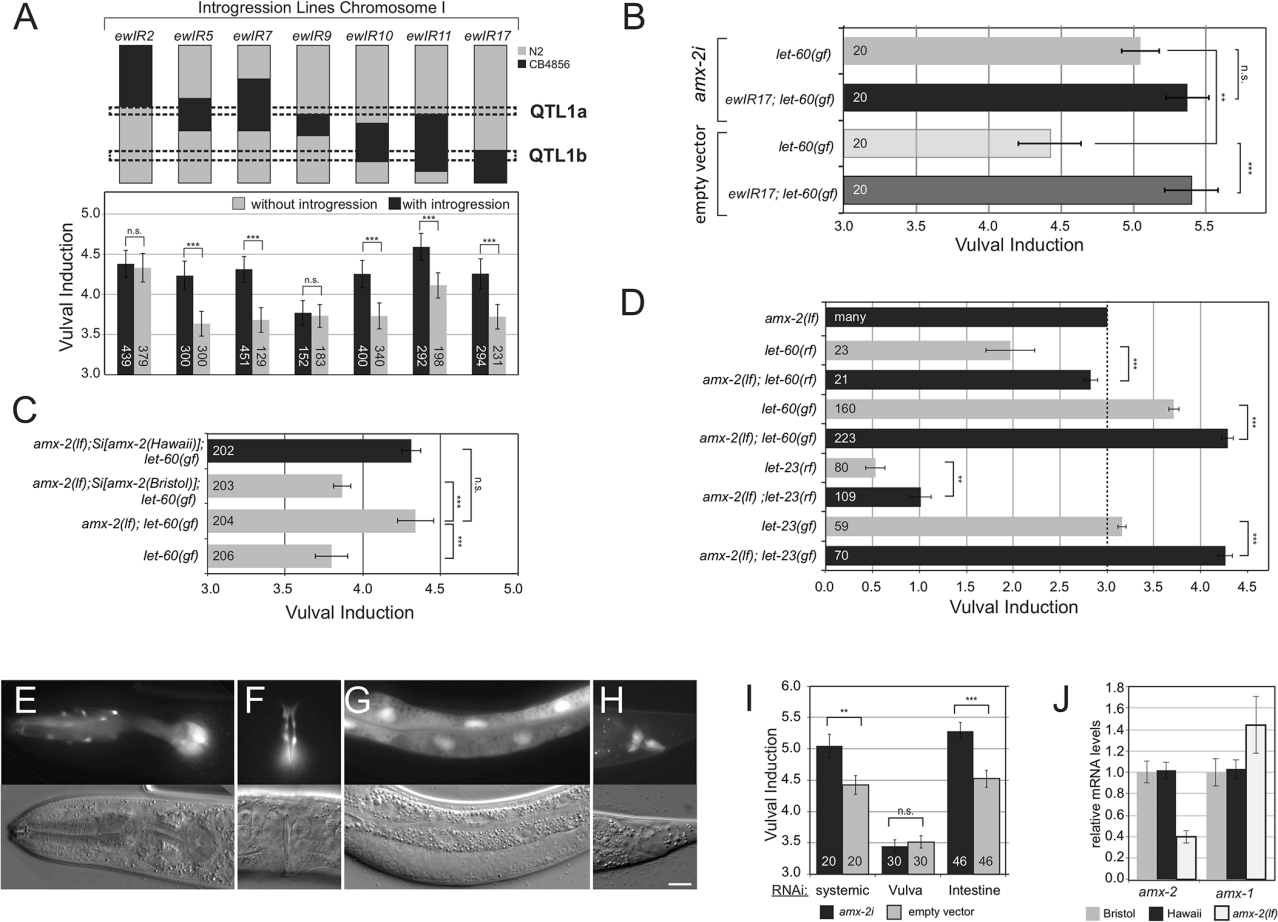
AMX-2 negatively regulates RAS/MAPK signaling. **(A)** Fine-mapping of QTL1 with ILs. For each IL, the regions containing the Hawaii (black) genome in the Bristol (grey) background are indicated, and the corresponding VIs are plotted below. Black columns indicate the average VI of three independent lines carrying an introgression and gray columns the average VI of three sibling lines without introgression. Dashed boxes indicate the QTL1a and QTL1b sub-regions. **(B)** Allele-specific effects of *amx-2* RNAi compared to empty vector controls. **(C)** Two copies of Bristol but not Hawaii *amx-2* rescue the increased VI of *amx-2(ok1235); let-60(n1046gf)* double mutants. **(D)** Epistasis analysis of *amx-2(ok1235)*. The dashed line indicates the wild-type VI of 3. **(E-H)** Expression pattern of a transcriptional *P*_amx-2_::*gfp* reporter in the pharynx and head neurons **(E)**, the adult vulva **(F)**, the intestine **(G)** and some rectal cells **(H)** of L4 larvae. The scale bar is 10μm. **(I)** Tissue-specific *amx-2* RNAi. Knock-down in the intestine but not the vulval cells increases the VI of *let-60(n1046gf)* mutants **(J)** Quantitative PCR of *amx-2* and *amx-1*. Expression levels were normalized to the N2 wild-type Bristol strain. Error bars in **(A)** to **(I)** indicate the standard error of the mean and in **(J)** the standard deviation measured in three independent experiments. The numbers of animals scored are shown inside the columns. *** indicates p<0.001, ** p<0.01, *<0.05 and n.s. p>0.1 in a Student’s t-test.

To identify polymorphic modifiers of the RAS/MAPK pathway, we generated a set of 228 “mutation included recombinant inbred lines” (miRILs) between the Bristol strain MT2124 that carries the activating *let-60 ras(n1046gf)* mutation [7] and the Hawaiian CB4856 strain (**Fig 1B**). Since small genetic variations are efficiently buffered in a wild-type genome [13,14], the inclusion of the *let-60(gf)* allele created a sensitized genetic background, allowing us to identify genetic modifiers that increase or decrease RAS/MAPK signaling. After 10 generations of inbreeding and genotyping using fragment length polymorphisms (FLPs) [15], 173 of the miRILs homozygous for the *n1046* allele were used for further analysis (**Fig 1C**, top) (see Materials and Methods for details on genotyping and the selection of informative miRILs). In addition, we quantified RAS/MAPK signaling output in each of these miRILs by measuring the VI of at least 20 animals. While the *let-60(gf)* allele in the Bristol background exhibits a VI of 3.7±0.06 (n = 100), the VIs of the miRILs varied between 3.0 and 5.7 (**Fig 1C**, bottom). Quantitative trait loci (QTL) mapping [14] identified at least three loci on chromosomes I (QTL1), II (QTL 2) and V (QTL 3) above the threshold LOD score of 3 that are associated with variation in the VI (**Fig 1D**). For QTL1, the Bristol genotype is associated with a decreased VI, while for QTL2 and QTL3 the Bristol genotype is associated with an increased VI (**S1 Fig**). To estimate the effect size of each QTL and explore how the QTLs affect the VI when combined, we used two mapping models, one where the QTLs have additive effects and another one where they show an interaction (**S1 Table**). This analysis did not detect any significant interactions between the QTLs. Since the *let-60(gf)* mutation maps to chromosome IV, our approach did not permit us to identify QTLs on this chromosome. Moreover, the genetic incompatibility between the Bristol and Hawaii genomes caused by the *zeel-1* and *peel-1* loci on the left arm of chromosome I may have prevented the detection of QTLs in this region [16]. To confirm and refine the mapping of the detected QTLs, introgression lines (ILs) carrying defined segments of the Hawaii genome in the QTL regions of interest were crossed to the *let-60(gf)* Bristol strain [17]. Lines homozygous for the introgressions and the *let-60(gf)* mutation were compared to sibling lines without introgressions to identify those introgressions that cause significant differences in the VI (see Materials and Methods). The results for the fine mapping of QTL1 are shown in **Fig 2A** and for all QTLs in **S2 Fig** IL mapping revealed that QTL1 is composed of two adjacent QTLs, termed 1a and 1b, and that QTL1b maps to an interval of 1.43 Mbp containing 142 polymorphic genes (**Fig 2A**). Through this approach, we have identified several regions in the *C*. *elegans* genome that contain modifiers of the RAS/MAPK pathway. Notably, for QTL1a and QTL1b the Bristol genotype caused reduced RAS/MAPK activity, while for QTL2 and QTL3 the Bristol background increased RAS/MAPK activity.

### The polymorphic *amx-2* gene negatively regulates RAS/MAPK signaling

Since the QTL1b region does not contain any known regulators of RAS/MAPK signaling, we performed RNAi knockdown of 107 of the 142 genes in this region in *let-60(gf)* single mutants as well as in *let-60(gf)* mutants carrying the *ewIR17* introgression, which spans QTL1b. We envisioned two possible scenarios that are not mutually exclusive: (1) The QTL1b region in the Bristol strain may contain a negative regulator of RAS/MAPK signaling that is inactive or weakly active in the Hawaii background. (2) The Hawaii background may contain a positive regulator of RAS/MAPK signaling that is inactive or weakly active in the Bristol background. We thus screened for candidates exhibiting allele-specific RNAi effects (**S2 Table**). Note that when grown on the *E*.*coli* strain HT115 that is commonly used in RNAi feeding experiments [18], the *let-60(n1046)* allele exhibits an increased VI compared to animals grown on standard OP50 bacteria [19]. Knockdown of five genes significantly increased the VI in the *let-60(gf)* but not in the *ewIR17; let-60(gf)* background, defining potential negative regulators of RAS/MAPK signaling that are active in the Bristol background (highlighted in green in **S2 Table**), whereas knockdown of ten genes reduced the VI in the *ewIR17; let-60(gf)* but not in the *let-60(gf)* background, defining potential positive regulators active in the Hawaii background (highlighted in blue in **S2 Table**). These data suggested that the QTL1b region is oligogenic, containing several polymorphic modifiers of RAS/MAPK signaling. Of particular interest was the *amx-2* gene because it fulfilled the criteria of a polymorphic negative regulator of RAS/MAPK signaling acting in the Bristol strain, but being inactive in the Hawaii strain. *amx-2* RNAi had no significant effect on the *ewIR17; let-60(gf)* background, but *amx-2* RNAi caused a robust increase in the VI of *let-60(gf)* mutants (**Fig 2B**). Furthermore, the *amx-2(ok1235)* deletion mutant, which most likely represents a null allele (www.wormbase.org), increased the VI of *let-60(gf)* mutants in the Bristol background (**Fig 2C**). To individually assess the activities of the Bristol and Hawaii *amx-2* variants, we generated single-copy insertions on chromosome II [20] of a 7.8 kb genomic fragment spanning the *amx-2* locus that was isolated either from the Bristol or the Hawaii genome. These single-copy transgenes were then introduced (homozygously) into the *amx-2(lf); let-60(gf)* background. Insertion of the Bristol but not the Hawaii *amx-2* variant reduced the VI of *amx-2(lf); let-60(gf)* double mutants to the value observed in *let-60(gf)* single mutants (**Fig 2C**). These results confirmed the different physiological activities of the two *amx-2* variants. In addition, *amx-2(lf)* partially suppressed the Vulvaless phenotype caused by reduction-of-function mutations in *let-60 ras* [7] or the EGFR homolog *let-23* [10] and enhanced the Multivulva phenotype of the *let-23* gain-of-function mutation *sa62* [21] (**Fig 2D**). We thus conclude that the Bristol variant of the *amx-2* gene inhibits RAS/MAPK signaling in the VPCs.

### *amx-2* in intestinal cells inhibits RAS/MAPK signaling cell non-autonomously

To determine the site of *amx-2* action, we generated transcriptional *P*_*amx-2*_::*gfp* reporters. *amx-2* was expressed in head neurons, the intestine and in a subset of cells of the rectum and in the adult vulva (**Fig 2E–2H**). However, we did not observe any *amx-2* expression in the VPCs during vulval induction, though *amx-2* reporter levels could be below the detection limit. Since neurons have a low sensitivity to RNAi [22], yet *amx-2i* efficiently phenocopied the *amx-2(lf)* phenotype, we suspected that *amx-2* might act in intestinal cells, where we detected strongest expression. Intestine-specific *amx-2* RNAi using an *rde-1(lf); let-60(gf); P*_*elt-2*_::*rde-1(+)* strain [23] increased the VI to a similar degree as systemic RNAi, while vulva-specific RNAi using the *P*_*lin-31*_::*rde-1(+)* transgene [24] had no detectable effect, which is consistent with lack of detectable *amx-2* reporter expression in the VPCs (**Fig 2I**, note that the overall lower VI in the vulva-specific RNAi strain is due to the genetic background [24]). Taken together, AMX-2 most likely acts in the intestinal cells to negatively regulate RAS/MAPK signaling in the VPCs.

### Expression of *amx-1* is increased in *amx-2(lf)* mutants

To investigate a possible redundancy between the MAOA *amx-2* and the MAOB gene *amx-1*, we measured the transcript levels of *amx-2* and its paralog *amx-1* by quantitative real-time PCR. The abundance of *amx-2* and *amx-1* transcripts was not significantly different between the Bristol and Hawaii backgrounds (**Fig 2J**). However, *amx-2* transcript levels were around 60% decreased and *amx-1* levels around 40% increased in *amx-2(lf)* mutants. Possibly, the elevated *amx-1* expression can partially compensate for a loss of *amx-2* expression.

### The 5-HT metabolite 5-HIAA acts as systemic inhibitor of RAS/MAPK signaling

**Fig 3:**
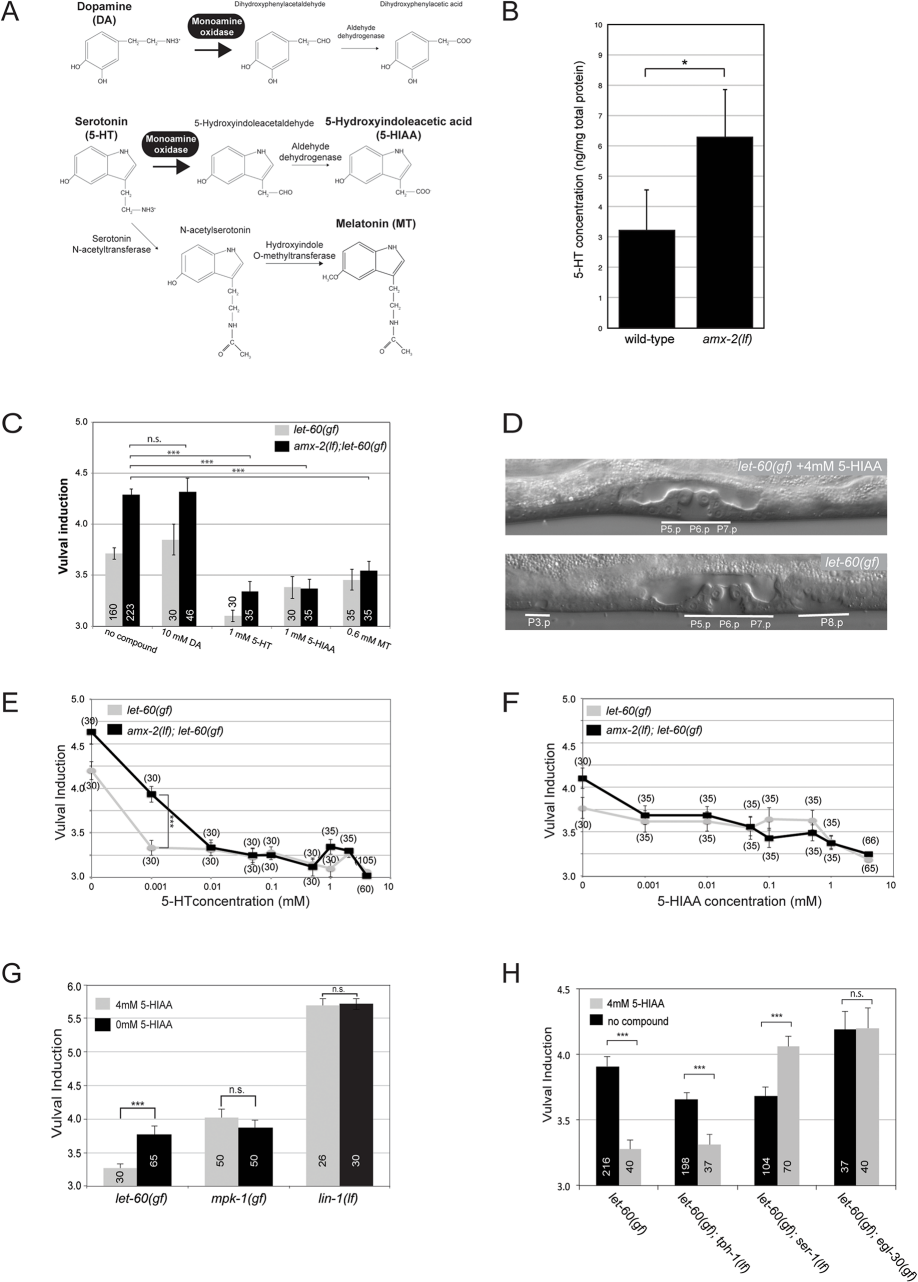
Systemic inhibition of RAS/MAPK signaling by Serotonin and its metabolites. **(A)** Function of MAOA in DA and 5-HT degradation. **(B)** 5-HT levels in total extracts of wild-type and *amx-2(ok1235)* animals. **(C)** Effect of DA, 5-HT and its metabolites on the VI of *let-60(n1046gf)* single and *amx-2(ok1235); let-60(n1046gf)* double mutants. **(D)** Examples of (top) a 5-HIAA treated and (bottom) an untreated *let-60(n1046gf)* L4 larva. The normal vulva and the ectopically induced cells are underlined. **(E)** Dose-dependent reduction of the VI by 5-HT and **(F)** 5-HIAA treatments. Note in **(E)** the different sensitivities of the two strains to 1μM 5-HT. **(G)** Effect of 5-HIAA on mutations activating the EGFR/RAS/MAPK pathway at different levels. **(H)** Resistance of some 5-HT pathway mutants to 5-HIAA treatment. Error bars indicate the standard error of the mean. The numbers of animals scored are indicated in brackets or inside the columns. *** indicates p<0.001, ** p<0.01, and n.s. p>0.1 in a Student’s t-test.

*amx-2* encodes a member of the mitochondrial monoamine oxidase (MAO) family [25]. Sequence alignments of the catalytic domains of different MAOs indicated that AMX-2 is most closely related to the ancestor of the mammalian MAOA, MAOB and L-amino oxidases (**S3 Fig**). The Hawaii AMX-2 variant possesses two coding polymorphisms in the catalytic domain (V410I and N461S) and another four in the C-terminal region (R521G, T532S, N535S and L617P) (**S4 Fig**). MAOs are key enzymes in the degradation of the neurotransmitters 5-HT and DA (**Fig 3A**) [12]. The products of the DA and 5-HT deamination reactions, 3,4-dihydroxyphenylacetaldehyde and 5-hydroxyindole-acetaldehyde respectively, are further oxidized by aldehyde dehydrogenases into 3,4-dihydroxyphenylacetic acid and 5-hydroxyindoleacetic acid (5-HIAA), which in humans are secreted through the kidneys (**Fig 3A**) [26]. Consistent with the predicted function of AMX-2 in degrading 5-HT, total extracts of *amx-2(lf)* worms contained elevated levels of 5-HT when compared to wild-type extracts (**Fig 3B**).

We thus investigated if AMX-2 inhibits RAS/MAPK signaling by controlling the levels of DA, 5-HT or their metabolites. The addition of 10mM DA to the growth medium had no significant effect on the VI of *let-60(gf)* single or *amx-2(lf); let-60(gf)* double mutants (**Fig 3C**). However, 1mM 5-HT as well as 1mM of the 5-HT metabolite 5-HIAA caused a strong reduction of the VI in both backgrounds and a suppression of the Multivulva phenotype (**Fig 3C and 3D**). Addition of 0.6mM melatonin (MT), another 5-HT metabolite (**Fig 3A**), had a slightly weaker yet significant effect on the VI (**Fig 3C**). We conclude that the 5-HT metabolites, in particular 5-HIAA, inhibit RAS/MAPK signaling. To test the sensitivity of the RAS/MAPK pathway to 5-HT and 5-HIAA, we performed dose-response experiments in the presence and absence of *amx-2*. For both compounds, the maximum reduction of the VI was observed at concentrations above 1mM (**Fig 3E and 3F**). However, *let-60(gf)* single mutants displayed a higher sensitivity to low concentrations (1μM) of 5-HT than *amx-2(lf); let-60(gf)* double mutants, while the effects of 5-HIAA were independent of the *amx-2* genotype. Overall, 5-HT exerted a slightly stronger effect than 5-HIAA, suggesting that additional 5-HT metabolites besides 5-HIAA may inhibit RAS/MAPK signaling.

To determine at which step 5-HIAA regulates the RAS/MAPK pathway, we examined a strain expressing an activated form of the MAPK MPK-1 along with the MAPKK MEK-2 [27] (*mpk-1(gf)*). Application of 4mM 5-HIAA did not alter the VI of *mpk-1(gf)* mutants (**Fig 3G**). Also, 5-HIAA did not affect a *lf* mutation in *lin-1*, which encodes an ETS family transcription factor that represses vulval induction downstream of MPK-1 [28] (**Fig 3G**). Taken together, these results indicate that 5-HIAA inhibits RAS/MAPK signaling upstream of MPK-1.

### 5-HIAA acts via the 5-HT receptor SER-1 and the EGL-30 Gqα pathway

We further characterized the inhibitory effect of 5-HIAA on the RAS/MAPK pathway by testing mutants in the 5-HT pathway for their response to 5-HIAA treatment. A mutation in the tryptophan hydroxylase gene *tph-1*, which is essential for 5-HT biosynthesis [29], slightly reduced the VI in l*et-60(n1046gf)* animals in the absence of 5-HIAA (**Fig 3H**). However, treatment of *tph-1(lf); let-60(n1046gf)* double mutants with 4mM 5-HIAA further reduced the VI, indicating that 5-HIAA acts in the absence of endogenous 5-HT and hence does not compete with 5-HT. By contrast, the VI of *let-60(n1046gf)* animals carrying a mutation in the 5-HT receptor gene *ser-1* [30] was not reduced by 5-HIAA treatment. Surprisingly, the VI of *let-60(gf); ser-1(lf)* double mutants was even increased after 5-HIAA treatment. Moreover, a gain-of-function mutation in *egl-30*, which encodes a Gqα protein acting in the 5-HT pathway [31], rendered *let-60(n1046gf)* mutants resistant to 5-HIAA and caused a slight increase of the VI in untreated animals (**Fig 3H**). Since the SER-1/EGL-30 pathway plays an essential role in 5-HT stimulated egg laying [30], we tested the effects of 5-HIAA on the egg laying rate with and without 5-HT stimulation. While 5-HIAA treatment alone caused a slight reduction in the egg laying rate, 5-HIAA did not significantly compete with the 5-HT stimulated increase in egg laying (**S5 Fig**). We conclude that 5-HIAA acts via the SER-1 receptor and the downstream EGL-30 Gqα signaling pathway to repress RAS/MAPK activity. However, the inhibitory effect of 5-HIAA is independent of 5-HT activity.

### 5-HIAA attenuates RAS/MAPK signaling in multiple organs of *C*. *elegans*

**Fig 4:**
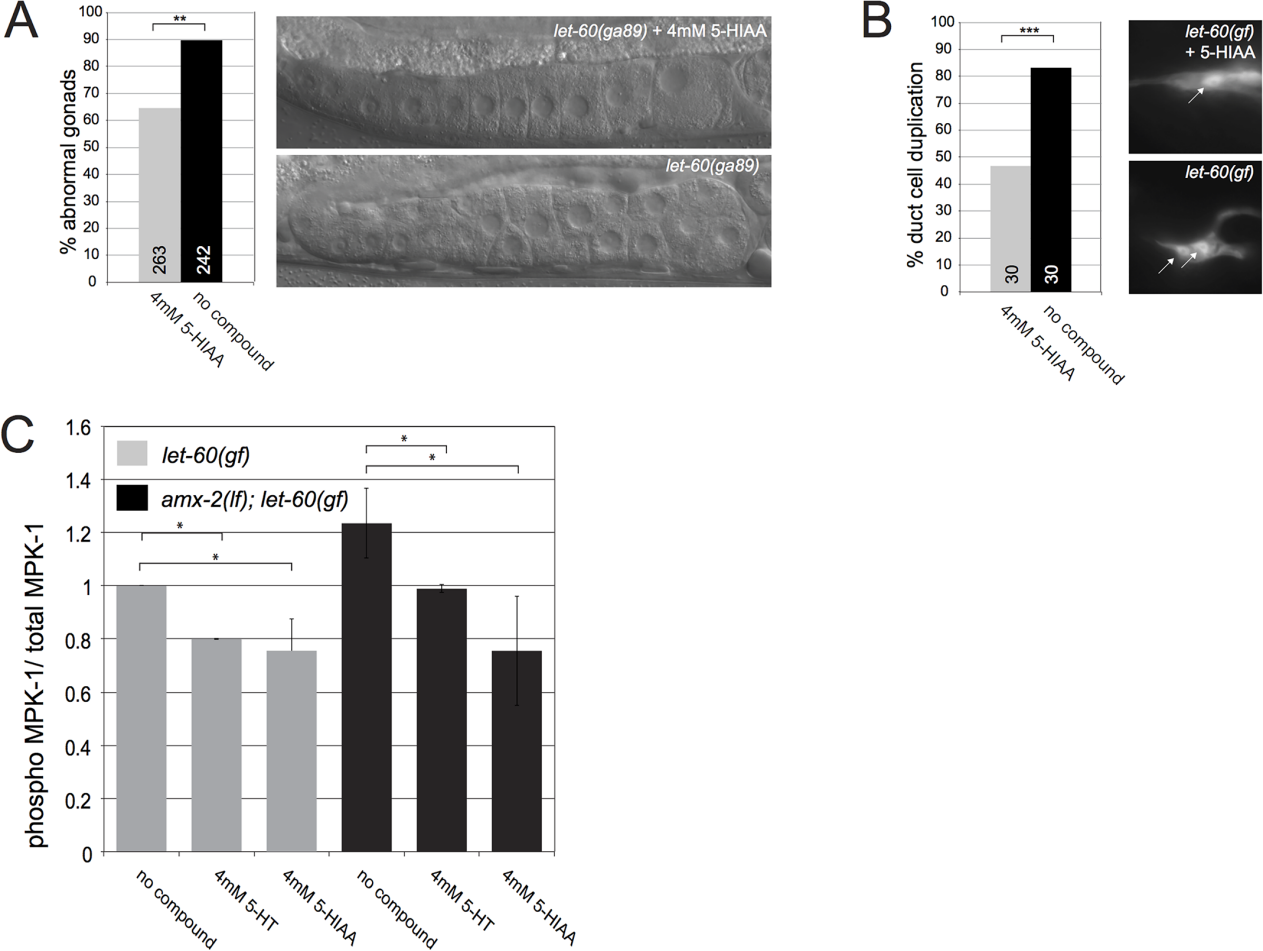
5-HIAA inhibits RAS/MAPK signaling and MPK-1 phosphorylation in multiple organs of *C*. *elegans*. **(A)** Partial suppression of the germline defect in *let-60(ga89ts)* mutants treated with 5-HIAA and grown at 25°C. The images show the gonads of 5-HIAA treated (top) and untreated (bottom) young adults. Note the regularly stacked oocytes in 5-HIAA treated and the irregularly stacked and smaller oocytes in untreated animals. **(B)** Partial suppression of the duct cell duplication phenotype in *let-60(n1046gf)* mutants by 5-HIAA. The images show the single duct cell in a 5-HIAA treated *let-60(n1046gf)* L4 larva (top) and the two duct cells in an untreated larva (bottom). The arrows point at the nuclei of the duct cells expressing the *lin-48*::*gfp* marker. **(C)** MPK-1 phosphorylation in total extracts of *let-60(n1046gf)* single and *amx-2(ok1235); let-60(n1046gf)* double mutant larvae treated with 4mM 5-HT or 5-HIAA. The ratios of phosphoMPK-1 to total MPK-1 levels were determined in three independent experiments as described in [11] and Materials and Methods. Values were normalized to the ratios in untreated animals. The numbers of animals scored are indicated in brackets or inside the columns. *** indicates p<0.001 and ** p<0.01 in a Student’s t-test

Besides the VPCs, RAS/MAPK signaling is required in several other organs of *C*. *elegans* [4]. Hence, *let-60(gf)* mutants exhibit multiple defects besides a Muv phenotype. For example, the temperature-sensitive *let-60(ga89gf)* allele causes accelerated exit of meiotic germ cells from the pachytene stage, resulting in the accumulation of many immature oocytes in the proximal gonad arm at the restrictive temperature [32,33] (**Fig 4A**). Moreover, *let-60(n1046gf)* mutants frequently contain two duct cells expressing the *lin-48*::*gfp* marker [34] (**Fig 4B**). Treatment with 4mM 5-HIAA partially suppressed the *let-60(gf)* phenotypes both in the germ line and the duct cell (**Fig 4A and 4B**). To measure the global effect of 5-HT and 5-HIAA treatment on MAPK activation, we quantified the levels of activated, phosphorylated MPK-1 in total extracts of L4 larvae [11]. Treatment with 5-HT and 5-HIAA caused a similar reduction in phospho-MPK-1 levels in *let-60(gf)* mutants. However, in the *amx-2(lf); let-60(gf)* background 5-HIAA exerted a stronger effect than 5-HT (**Fig 4C**). Thus, 5-HIAA supplemented into the culture medium exerts a systemic effect to inhibit RAS/MAPK signaling in different organs of *C*. *elegans*.

## Discussion

We have identified several genetic modifiers of the oncogenic RAS/MAPK signaling pathway by comparing miRILs derived from the backgrounds of two highly diverged *C*. *elegans* isolates. The two parental strains used in this study display a level of sequence divergence that is comparable to the genetic variation observed in the human population [35]. The genetic modifiers of RAS/MAPK signaling we identified through this quantitative approach could not have been found in conventional forward genetic screens, as each locus alone only exerts a minor effect. Interestingly, both genetic backgrounds analyzed contain QTLs that enhance (i.e. QTLs 2 and 3 for Bristol) as well as QTLs that reduce (i.e. QTL 1 for Bristol) the relative strength of RAS/MAPK signaling. Thus, each isogenic background may represent a balanced state exhibiting intermediate RAS/MAPK pathway activity thanks to the opposing effects of the different modifiers. The interplay of these modifiers may be necessary to keep the activity of the RAS/MAPK pathway within a certain range and avoid the detrimental effects caused by increased or reduced RAS/MAPK signaling.

The molecular characterization of one particular region (QTL 1b) identified the monoamine oxidase gene *amx-2* as a negative regulator of RAS/MAPK signaling in multiple organs of *C*. *elegans*. Though, the RNAi analysis of the QTL1b region indicated that this region contains possibly up to ten additional polymorphic modifiers of RAS/MAPK signaling besides *amx-2*. Single-copy gene insertion experiments [20] demonstrated that the Bristol variant can fully rescue an *amx-2* deletion allele, while insertion of the Hawaii locus had no significant effect in this assay, indicating that *amx-2* activity in the Hawaii background is severely reduced or even absent.

The identification of a monoamine oxidase as a negative regulator of RAS/MAPK signaling was initially a surprising result, since MAOA is primarily known for its role in degrading neurotransmitters in the nervous system [12]. However, we observed strong AMX-2 expression in non-neuronal tissues, especially in the intestinal cells. The 5-HT metabolites such as 5-HIAA that result from AMX-2 catalysis are likely to be released into the body cavity in order to modulate RAS/MAPK signaling in distant organs. Such a globally acting regulatory mechanism may be useful to rapidly adjust RAS/MAPK signaling in response to changing environmental conditions, after food intake and to adapt the speed of reproduction [36]. Epistasis analysis by applying exogenous 5-HIAA points at a step downstream of RAS and upstream of MAPK that is repressed by 5-HIAA. Hence, 5-HIAA may simultaneously repress the RAS/MAPK pathway activated by various receptor tyrosine kinases in different tissues [4]. The observation that 5-HT exerts an inhibitory effect even in *amx-2(0)* mutants may be explained by the presence of additional redundant MAOs, notably AMX-1, and by spontaneous oxidation of 5-HT. Our epistasis analysis further indicates that 5-HIAA acts via the SER-1 receptor, which activates the EGL-30 Gqα signaling pathway [31]. One possible scenario is that 5-HIAA and 5-HT exert opposing effects on SER-1, such that the balance between 5-HT and 5-HIAA levels determines the strength of EGL-30 activation, which in turn promotes RAS/MAPK signaling. In line with this model, Moghal et al. [37] have previously shown that *egl-30* signaling in neuronal cells positively regulates vulval induction under different environmental conditions.

The role of 5-HT as a neurotransmitter in the mammalian nervous system is well documented [12]. However, over 90% of the 5-HT in the human body is found outside of the nervous system, especially in enterochromaffin cells of the intestine [38]. Remarkably, Rybaczyk et al. [39] reported that the expression of the human 5-HT degrading enzyme MAOA, the closest AMX-2 homolog, is consistently down-regulated across many human tumor types. The functional implications and mechanisms of reduced MAOA expression in cancer cells are unclear. Our findings that systemic application of the 5-HT metabolite 5-HIAA globally inhibits RAS/MAPK signaling may explain the physiological consequences of MAOA down-regulation. Tumors expressing low levels of MAOA may generate less oncostatic 5-HIAA and at the same time contain higher levels of 5-HT, which can promote tumor growth and survival via cross-talk to the RAS/MAPK pathway [40,41]. Thus, MAOA levels may set a global threshold for the activation of the RAS/MAPK cascade by different extracellular signals. To our knowledge, 5-HIAA is the first endogenous small molecule that acts as a systemic inhibitor of the RAS/MAPK pathway.

## Materials and Methods

### General methods and strains used

Strains were maintained on NGM agar seeded with OP50 bacteria at 20°C [42], unless otherwise stated. *C*. *elegans* Bristol refers to the wild-type N2 strain and Hawaii to CB4856 [8]. Transgenic lines were generated as described in [32,43].

### Mutations used

**LG I:** *amx-2(ok1235)*, *egl-30(tg26)* [31]; **LG II:** *let-23(sa62)*[21], *let-23(sy1)* [44], *tph-1(n4622)* [29]; **LG IV:** *let-60(ga89)* [32], *let-60(n1046)* [7], *let-60(n2021)* [7], *lin-1(n304)* [45]; **LG V:** *rde-1(ne219)* [46] **LG X:** *ser-1(ok345)* [47]. **Transgenic strains:** *rde-1(ne219); duIs[P*_*elt-2*_::*rde-1(+); pRF4]* [23], *let-60(n1046); rde-1(ne209); zhEx418[P*_*lin-31*_::*rde-1(+); myo-2*::*mCherry]* [24], *gaIS37[HS-mpk-1*, *dmek]* [27], *let-60(n1046); saIS14[lin-48p*::*gfp]* [34], *zhEx533[P*_*amx-2*_::*gfp*, *P*_*myo-2*_::*mcherry]*, *amx-2(ok1235); zhSi73[amx-2 Bristol]; let-60(n1046gf); zhSi74[amx-2 Hawaii]; let-60(n1046gf)* (all this study).

### Generation and genotyping of *let-60(n1046)* miRILs and Ils

miRILs were generated by crossing CB4856 males with MT2124(*let-60(n1046)*) hermaphrodites. In the F2 generation, lines homozygous for the *n1046* allele were singled out and allowed to self-fertilize for 10 more generations to reach homozygosity by random cloning of individuals. At generation F12, lines were regarded as isogenic and frozen for long-term storage. All 228 miRIL lines were genotyped with the following 72 FLP markers as described in [15]: *zh1-17; zh1-10a; zh1-07; zh1-18a; zh1-03; zh1-27; zh1-34; zh1-01;zh1-23; zh1-15; zh1-08; zh1-06; zh2-04a; zh2-16; zh2-07; zh2-13; zh2-19; zh2-02; zh2-20; zh2-25; zh2-27; zh2-09; zh2-10; zh2-12; zh3-17a; zh3-07; zh3-06; zh3-08; zh3-28; zh3-15; zh3-04; zh3-02; zh3-05a; zh3-35; zh3-10a; zh3-11; zh3-13; zh4-04a; zh4-5; zh4-06; zh4-16; zh4-08; zh4-17; zh4-18; zh4-19; zh4-20; zh4-21; zh4-12; zh5-13; zh5-03a; zh5-14; zh5-05; zh5-16; zh5-17; zh5-18; zh5-11; zh5-12; zh5-08; zh5-21/22; zh5-09zhX-17; zhX-08; zhX-13; zhX-15; zhX-10; zhX-24; zhX-07; zhX-12; zhX-11; zhX-21a; zhX-06; zhX-23*. miRILs that contained a 100% Bristol genotype and miRILs lacking the *n1046* allele were excluded from further analysis, and miRIls with identical genotypes were combined. These criteria reduced the 228 initial miRILs to 173 informative lines. To generate ILs in the *n1046* background, the ewIR ILs from [14,17] were crossed with the MT2124(*let-60(n1046)*) mutant. For the exact breakpoints of the ILs used, see [17]. FLP mapping with 7 to 8 markers in the respective regions was used to identify and verify lines homozygous for the introgressions and exclude the presence of additional recombination events. Control siblings without an introgression were isolated in parallel, and the multivulva phenotype was used to identify homozygous *let-60(n1046)* lines. To quantify the VI, at least three independent introgression lines were compared to three sibling lines containing the *let-60(n1046)* allele but no introgression.

### Phenotyping

To measure the VI, vulval induction was scored in L4 larvae using Nomarski optics as described [48], and the average number of induced VPCs per animal was calculated. The duct cell duplication phenotype was scored using the *lin-48*::*gfp* marker to visualize the duct cells using fluorescence microscopy [34]. The oocyte maturation phenotype was scored in 2 day old adults under Nomarski optics microscopy.

### QTL mapping and data storage

QTL mapping was performed using a single marker model on the per miRIL averages. Significance threshold was estimated using 1000 permutations [14]. All QTL data, phenotypes, QTL profiles and genotypes are stored in www.WormQTL.org [49].

### RNA interference

Gene knock-down was carried out using RNAi feeding according to [18]. For intestine-specific RNAi, OLB11(*rde-1(ne219); duIs[P*_*elt-2*_::*rde-1(+); pRF4]*) [23] was crossed with the MT2124(*let-60(n1046)*) strain. For vulva-specific RNAi, the strain AH2927(*rde-1(ne219lf); let-60(n1046); zhEx418[Plin-31*::*rde-1; Pmyo-2*::*mcherry]*) described in [24] was used.

### Generation of single-copy insertion lines

A 7.8 kb genomic fragment spanning the entire *amx-2* locus was amplified with the primers OTS123 (GATTTTGGAGAAGAAACGAGGG) and OTS124 (ACTTCACTATGTTCCTCTACCG) using either Bristol or Hawaii genomic DNA as template and subcloned into the XhoI restriction site of pCFJ151 [20]. Single-copy insertions of the *amx-2 Bristol* and *amx-2 Hawaii* containing plasmids into the *ttTi5605* region on chromosome II were generated using the protocol by [20] to yield *zhSi73* and *zhSi74*, respectively. The insertions were verified by PCR amplification using primers flanking the insertion site before crossing them into the *amx-2(lf); let-60(gf)* background. For each genotype, at least three independent lines were scored.

### Transcriptional *amx-2* reporters

Primers OTS219 (AAA AGG ATC CTT AGG TTT ATT GCT GGA AAA AT) and OTS220 (AAA AGG ATC CCC TTA ACC AAA TTT CAT ACC C) were used to amplify 4kb of upstream promoter region. The PCR fragment was further cloned into the BamHI restriction site of pPD95.67 to generate a the *P*_*amx-2*_::*gfp* transcriptional reporter plasmid that was co-injected at 50ng/μl with 2.5ng/μl of the pharyngeal P_*myo-2*_::*mcherry* marker.

### Measurement of 5-HT levels

Animals were grown in 100ml liquid cultures and harvested by flotation on 50% sucrose. Worm pellets were resuspended in 2ml PBS buffer and lysed using a swing-mill homogenizer followed by high-speed centrifugation to remove insoluble debris. Total protein concentrations were measured in each sample using the amidoblack staining assay [50]. 5-HT levels were determined with an ELISA kit according to the manufacturer’s instructions (BA E-5900, Labor Diagnostika Nord) and normalized to the total protein concentrations in the extracts. The average 5-HT concentrations for each genotype were determined with two separate measurements, each done in triplicate using extracts obtained from two independently grown cultures.

### Treatment of *C*. *elegans* with 5-HT and its metabolites

Standard NGM plates were supplemented with the indicated concentrations of serotonin (5-HT) (H9523, Sigma), 5-Hydroxyindoleacetic acid (5-HIAA) (H8876, Sigma), dopamine (DA) (H8502, Sigma) or Melatonin (MT) (M5250 Sigma) and kept in dark at 4°C prior to use.

### Quantification of ERK phosphorylation

Phospho MPK-1 levels in total extracts of *C*. *elegans* L4 larvae were determined by Western blotting as described in [11]. As loading controls, total MPK-1 levels were quantified on parallel blots loaded with the same amounts of protein (20μg) from the identical samples. Protein bands were quantified using the integrated density function in ImageJ. The ratios of phospho-MPK-1 to total MPK-1 levels were calculated for each extract and normalized to the ratios in untreated controls. Antibodies used: anti-MAP Kinase (Sigma-Aldrich, M5670), anti-phosphoMAP Kinase, Activated (Diphosphorylated ERK-1&2, Sigma-Aldrich, M8159).

## Supporting Information

S1 Fig QTL effect sizes. In each of the panels showing chromosomes I through X, the QTL effect sizes were plotted along the chromosomal locations as shown in Fig 1D. Positive values indicate regions where the Bristol genotype increases and and negative values where the Bristol genotype decreases the VI.
(JPG)

S2 Fig Fine-mapping of the QTLs shown in Fig 1D with ILs. ILs covering the predicted QTL regions were chosen from [17] and crossed with the *let-60(n1046)* Bristol mutant. Significant differences in the VI indices between sibling lines with and without introgression were used to verify and further refine the different QTL regions. Several overlapping introgression lines allowed us to further narrow down the genomic intervals for further studies. The sizes and approximate positions of the ewIR introgressions are depicted below. For the exact locations of the breakpoints in each IL, see [17]. Error bars indicate the standard error of the mean, and *** indicates p<0.001 in a Student‘s t-test.
(JPG)

S3 Fig Sequence similarity between the catalytic domains of AMX-2 and mammalian monoamine oxidases. The catalytic domain of *C*. *elegans* AMX-2 (NP_493236) is most similar to mammalian MAOA (NP_000231), MAOB (AAH22494) and LAAO (NP_690863). The *C*. *elegans* genome encodes another five putative monoamine oxidase genes, *amx-1* (NP_497772.2), *amx-3* (NP_001256963), *hpo-15* (NP_504456.1), *lsd-1* (NP_510000) and *spr-5* (NP_493366.1), that are more distantly related to mammalian monoamine oxidases.
(JPG)

S4 Fig Coding polymorphisms between *C*. *elegans* Bristol and Hawaii AMX-2. Structure of the AMX-2 protein. The blue box indicates the conserved catalytic amine oxidase domain. Coding polymorphisms are found mainly in the C-terminal region of the protein (R521G, T532S, N535S and L617P). Only two non-synonymous polymorphisms (V410I and N461S) affect the catalytic amine oxidase domain.
(JPG)

S5 Fig Effects of 5-HT and 5-HIAA on the egg laying rate in liquid. Egg laying rates of one day-old adults were determined in liquid as described in [30]. For each genotype and condition, in total 48 animals were assayed in four independent experiments and the average egg laying rates per animal are shown. Error bars indicate the standard error of the mean, ** indicates p<0.01, * p<0.05 and n.s. p>0.1 in a Student’s t-test.
(JPG)

S1 Table Estimated QTL effect sizes in additive and interactive mapping models. (PDF)

S2 Table Results of the RNAi screen in the QTL1b region. Rows highlighted in green indicate allele-specific effects in the Bristol background, rows highlighted in blue indicate allele-specific effects in the Hawaii background, and rows highlighted in beige allele-independent effects. ^1^SE indicates the standard error of the mean. ^2^p-values compared to empty vector controls were determined with a Student’s t-test.
(PDF)
