# Supplementary figures and images for: Systemic Regulation of RAS/MAPK Signaling by the Serotonin Metabolite 5-HIAA
Source: PLoS Genet. 2015 May 15;11(5):e1005236. doi: 10.1371/journal.pgen.1005236 (PMC4433219; doi:10.1371/journal.pgen.1005236)

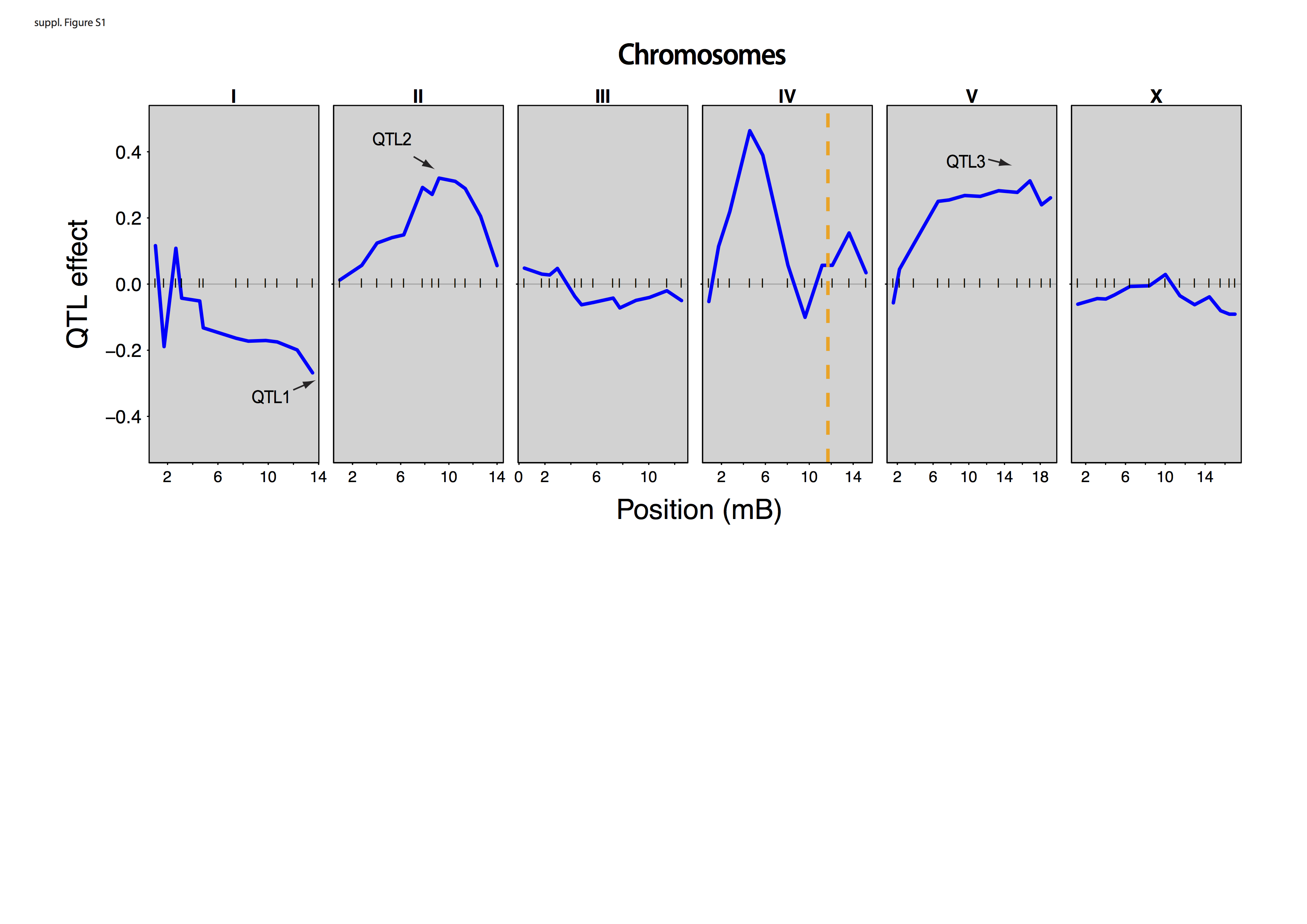

Supplement: S1 Fig — In each of the panels showing chromosomes I through X, the QTL effect sizes were plotted along the chromosomal locations as shown in Fig 1D. Positive values indicate regions where the Bristol genotype increases and and negative values where the Bristol genotype decreases the VI. (JPG) [file pgen.1005236.s001.jpg]

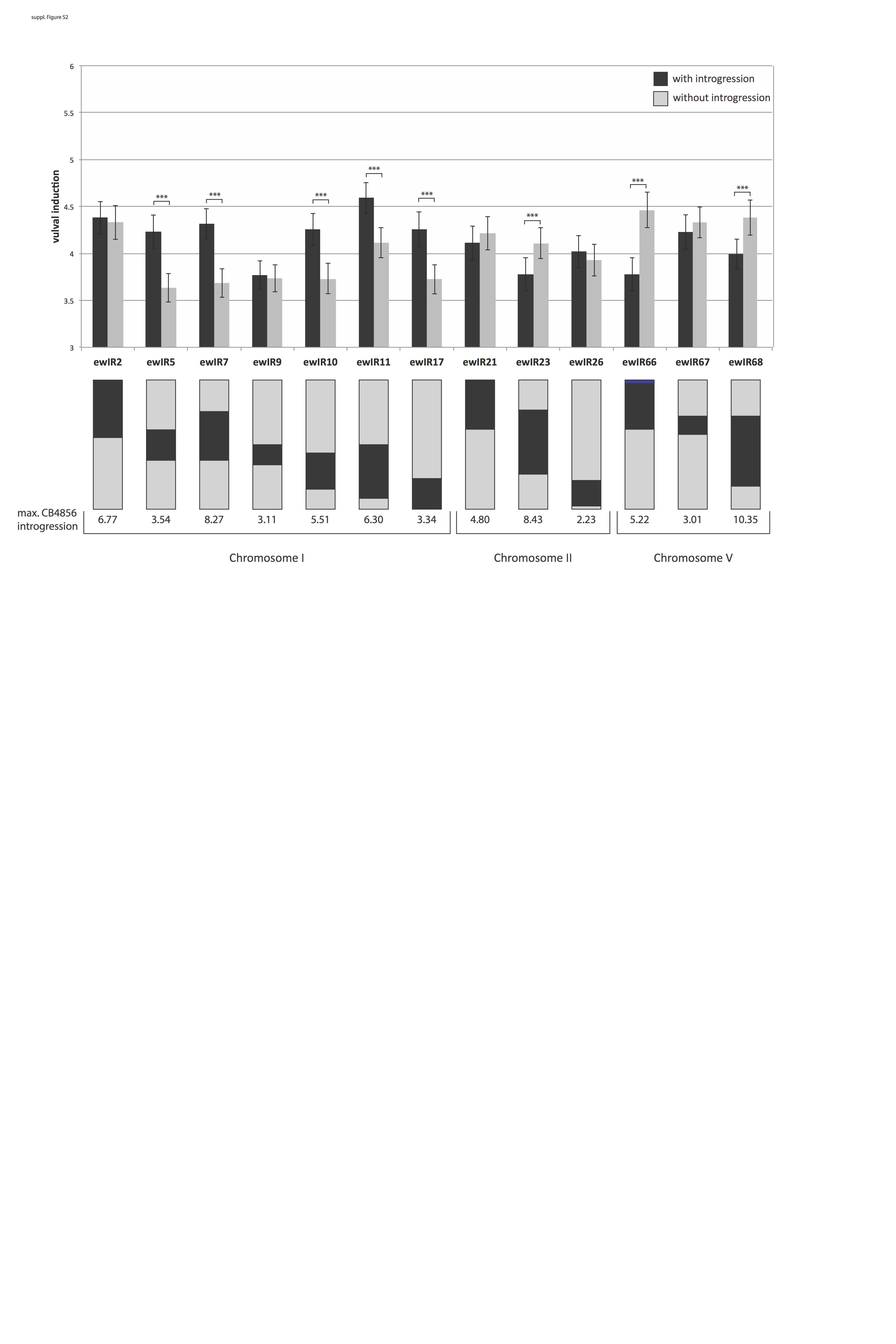

Supplement: S2 Fig — ILs covering the predicted QTL regions were chosen from [17] and crossed with the let-60(n1046) Bristol mutant. Significant differences in the VI indices between sibling lines with and without introgression were used to verify and further refine the different QTL regions. Several overlapping introgression lines allowed us to further narrow down the genomic intervals for further studies. The sizes and approximate positions of the ewIR introgressions are depicted below. For the exact locations of the breakpoints in each IL, see [17]. Error bars indicate the standard error of the mean, and *** indicates p<0.001 in a Student‘s t-test. (JPG) [file pgen.1005236.s002.jpg]

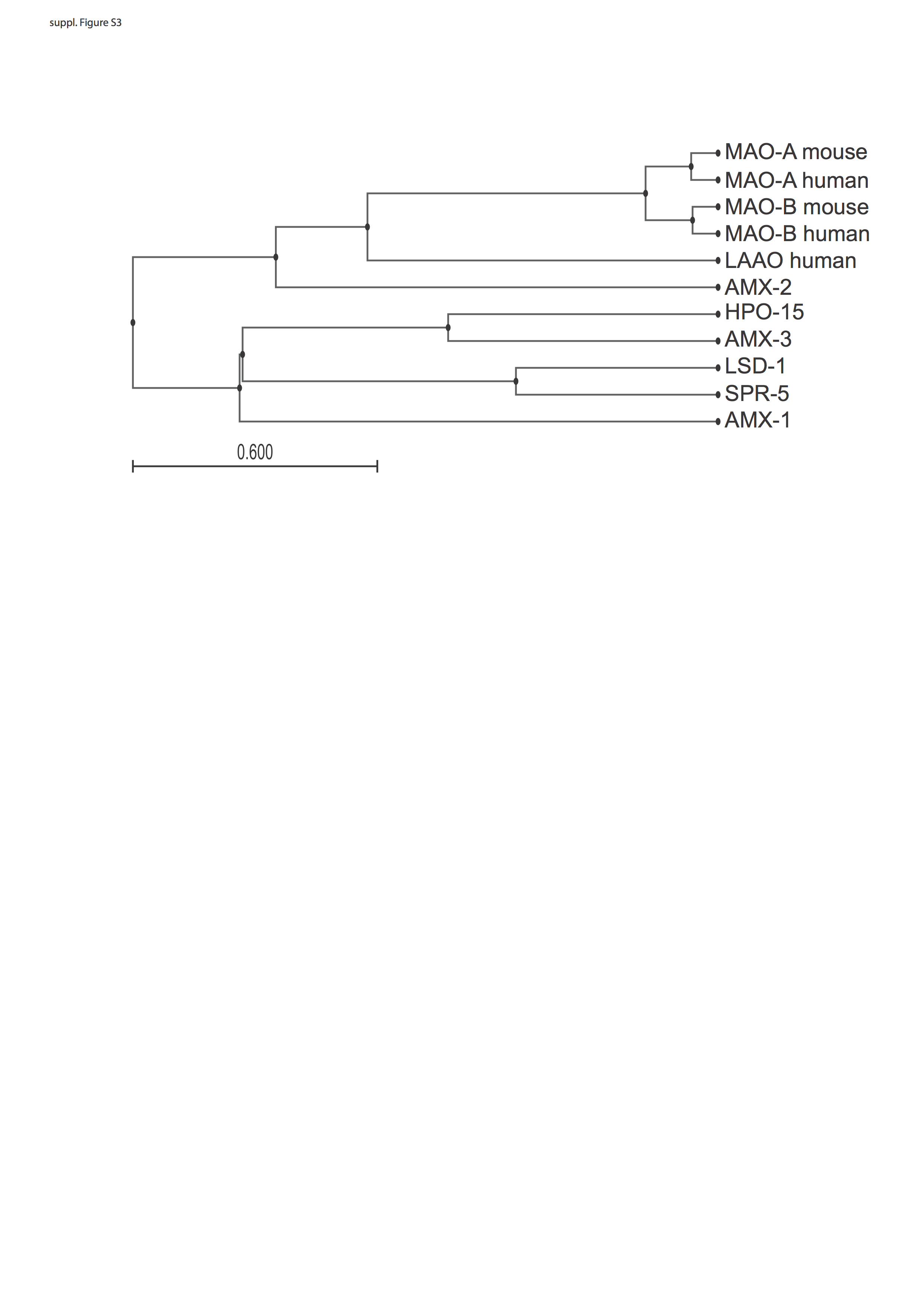

Supplement: S3 Fig — The catalytic domain of C. elegans AMX-2 (NP_493236) is most similar to mammalian MAOA (NP_000231), MAOB (AAH22494) and LAAO (NP_690863). The C. elegans genome encodes another five putative monoamine oxidase genes, amx-1 (NP_497772.2), amx-3 (NP_001256963), hpo-15 (NP_504456.1), lsd-1 (NP_510000) and spr-5 (NP_493366.1), that are more distantly related to mammalian monoamine oxidases. (JPG) [file pgen.1005236.s003.jpg]

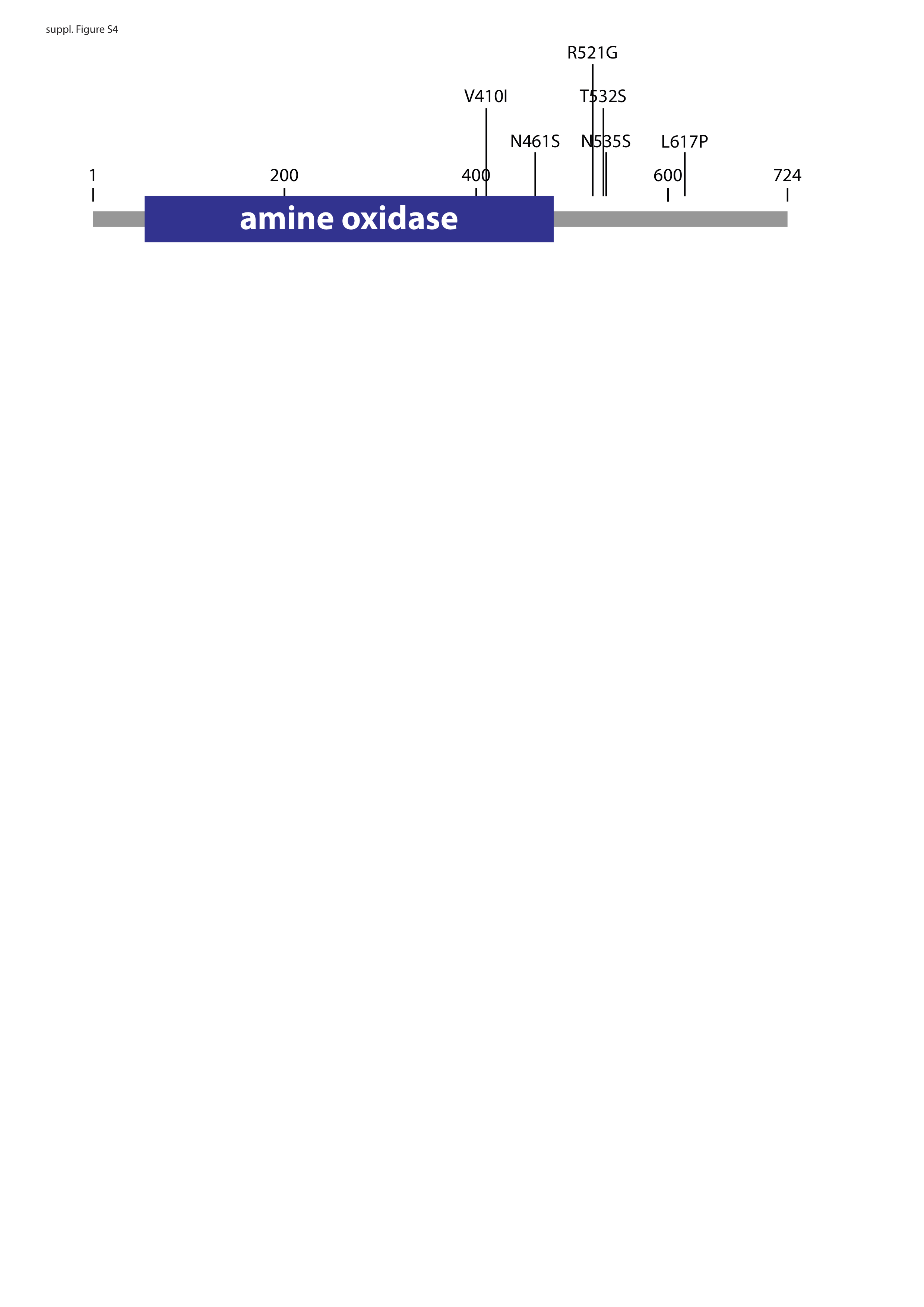

Supplement: S4 Fig — Structure of the AMX-2 protein. The blue box indicates the conserved catalytic amine oxidase domain. Coding polymorphisms are found mainly in the C-terminal region of the protein (R521G, T532S, N535S and L617P). Only two non-synonymous polymorphisms (V410I and N461S) affect the catalytic amine oxidase domain. (JPG) [file pgen.1005236.s004.jpg]

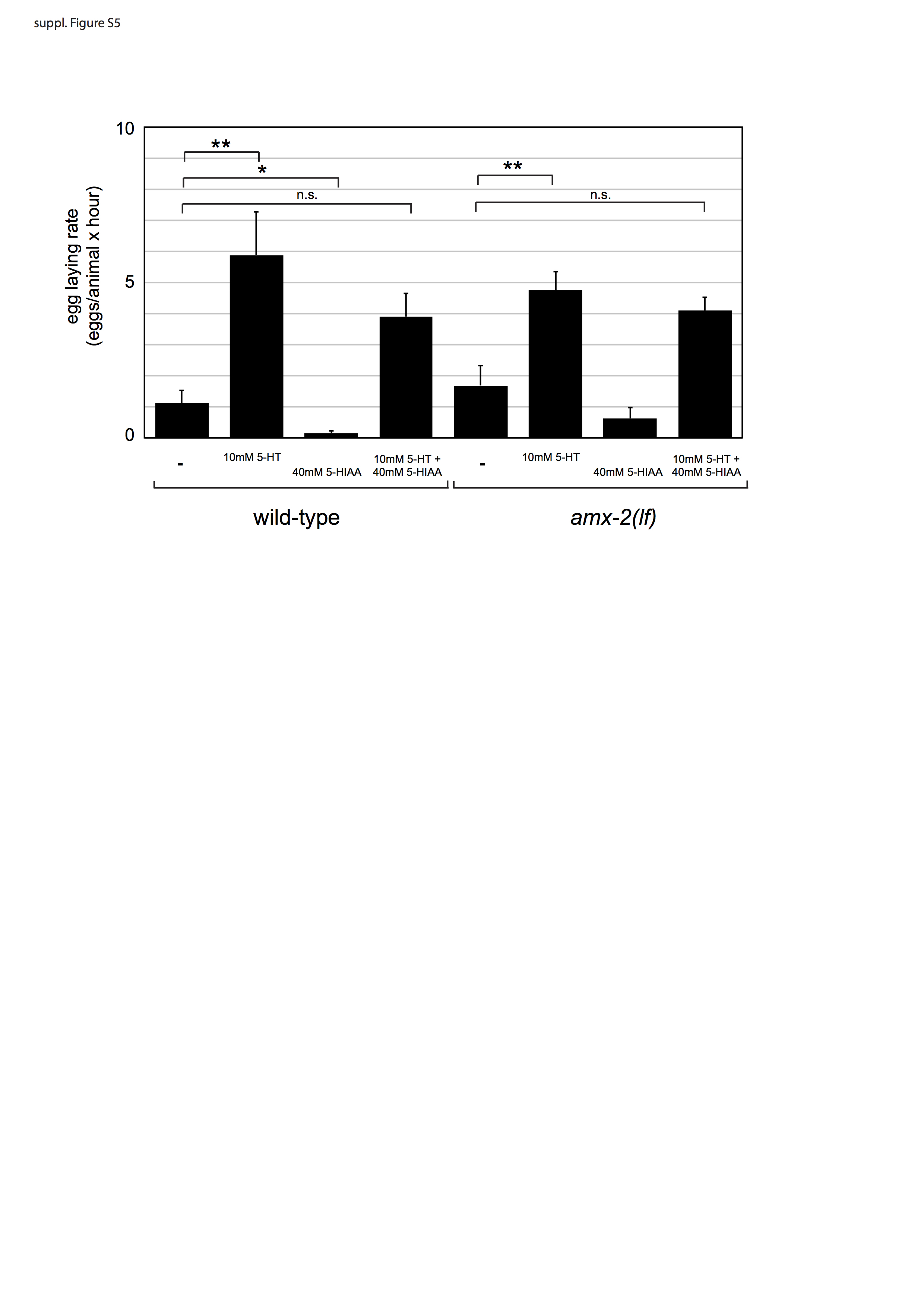

Supplement: S5 Fig — Egg laying rates of one day-old adults were determined in liquid as described in [30]. For each genotype and condition, in total 48 animals were assayed in four independent experiments and the average egg laying rates per animal are shown. Error bars indicate the standard error of the mean, ** indicates p<0.01, * p<0.05 and n.s. p>0.1 in a Student’s t-test. (JPG) [file pgen.1005236.s005.jpg]
