# Supplementary material for: Systemic Regulation of RAS/MAPK Signaling by the Serotonin Metabolite 5-HIAA
Source: PLoS Genet. 2015 May 15;11(5):e1005236. doi: 10.1371/journal.pgen.1005236 (PMC4433219; doi:10.1371/journal.pgen.1005236)
Supplement: S1 Table — (PDF) [file pgen.1005236.s006.pdf]

Table S1

Additive mapping model: mean VI ~ QTL1 + QTL2 + QTL3

QTL	Effect	p-value
1	-0.24	$4.3*10^{-6}$
2	0.27	$2.6*10^{-7}$
3	0.21	$4.3*10^{-5}$

Adjusted R-squared 0.36 ; p-value whole model: $2.5*10^{-16}$

Interaction mapping model: mean VI ~ QTL1 * QTL2 * QTL3

QTL	Effect	p-value
1	-0.23	$3.0*10^{-5}$
2	0.24	$1.0*10^{-5}$
3	0.20	$2.0*10^{-4}$
1*2	-0.07	0.17
1*3	-0.01	0.81
2*3	-0.03	0.52
1*2*3	-0.01	0.82

Adjusted R-squared 0.36 ; p-value whole model: $5.1*10^{-14}$
