# Supplementary material for: Systemic Regulation of RAS/MAPK Signaling by the Serotonin Metabolite 5-HIAA
Source: PLoS Genet. 2015 May 15;11(5):e1005236. doi: 10.1371/journal.pgen.1005236 (PMC4433219; doi:10.1371/journal.pgen.1005236)
Supplement: S2 Table — Rows highlighted in green indicate allele-specific effects in the Bristol background, rows highlighted in blue indicate allele-specific effects in the Hawaii background, and rows highlighted in beige allele-independent effects. 1SE indicates the standard error of the mean. 2p-values compared to empty vector controls were determined with a Student’s t-test. (PDF) [file pgen.1005236.s007.pdf]

Table S2

Gene Name	RNAi in:					
	let-60(gf)	SE[1]	p[2]	*ewIR17; let-60(gf)*	SE[1]	p[2]
E03H4.11	4.50	0.20		5.10	0.14	
C54C8.2	4.25	0.22		4.55	0.24	3.2E-03
C54C8.4	4.55	0.16		5.38	0.11	
glct-5	4.30	0.22		5.15	0.18	
T27F6.1	4.48	0.19		5.35	0.17	
pars-2	4.40	0.22		5.03	0.14	
K11D2.4	4.55	0.19		5.48	0.12	
W02D9.4	5.23	0.17	1.2E-03	5.08	0.20	
W02D9.9	4.53	0.19		4.75	0.19	
dlk-1	4.58	0.19		5.20	0.15	
F33E2.4	4.15	0.25		4.78	0.19	
F33E2.6	4.55	0.21		5.10	0.19	
T07D10.1	4.20	0.18		4.88	0.18	2.3E-02
ntr-1	4.90	0.21		5.20	0.15	
T07D10.3	4.03	0.23		5.00	0.11	2.6E-02
clec-103	4.68	0.13		5.35	0.16	
ndx-1	4.08	0.22		4.98	0.18	
T26E3.5	4.25	0.16		5.38	0.14	
T26E3.6	3.95	0.16	3.6E-02	4.73	0.19	6.1E-03
sra-25	4.55	0.21		5.08	0.15	
sra-17	4.98	0.17		5.65	0.16	
sra-20	3.58	0.12	9.0E-04	3.95	0.13	4.2E-03
sra-23	4.23	0.17		4.98	0.19	
*T06G6.5**	4.15	0.16		5.40	0.12	
T06G6.6	3.90	0.20	4.3E-02	5.23	0.18	
srw-88	3.38	0.10	1.2E-06	4.70	0.16	1.5E-03
pfd-3	3.98	0.19		4.60	0.18	7.5E-04
W02A11.1	3.83	0.15	7.7E-03	5.05	0.14	
vps-25	4.65	0.20		5.58	0.10	
toe-4	3.65	0.17	1.4E-03	4.38	0.20	1.4E-04
uba-2	4.70	0.17		5.38	0.18	
*bath-34**	4.10	0.16		4.85	0.21	2.9E-02
bath-35	4.18	0.22		5.05	0.14	
amx-2	5.05	0.18	1.2E-02	5.38	0.21	
B0019.2	3.95	0.21		5.30	0.16	
Y18D10A.1	4.28	0.20		5.08	0.14	
Y18D10A.2	3.68	0.16	1.5E-03	4.48	0.19	3.0E-04
ptr-17	4.65	0.22		5.13	0.14	
Y18D10A.8	4.45	0.17		4.48	0.19	
Y18D10A.9	3.58	0.16	8.9E-05	4.38	0.19	
clec-104	4.70	0.23		5.15	0.17	
clec-106	4.10	0.17		5.10	0.13	
pad-1	3.90	0.24		4.73	0.16	2.5E-03
F08A8.1	4.35	0.16		5.08	0.16	

Gene Name	RNAi in:					
	let-60(gf)	SE[1]	p[2]	*ewIR17; let-60(gf)*	SE[1]	p[2]
F08A8.4	4.25	0.20		5.50	0.14	
F08A8.5	3.93	0.18	3.9E-02	4.48	0.16	5.0E-05
fbxa-140	4.43	0.18		4.73	0.19	
C47B2.2	4.05	0.14		5.45	0.12	
gale-1	4.48	0.19		4.05	0.21	3.8E-02
selb-1	4.23	0.16		5.15	0.21	
prx-11	4.23	0.13		4.98	0.21	
smu-1	3.83	0.15	6.9E-03	4.75	0.24	2.2E-02
clec-108	4.65	0.17		5.28	0.19	
Y26D4A.8	3.93	0.20		5.68	0.13	
Y26D4A.9	4.38	0.18		5.48	0.11	
C17H1.4	4.75	0.21		5.68	0.09	
C17H1.7	4.90	0.17		5.23	0.14	
F22G12.5	4.85	0.17		5.30	0.14	
F17B5.1	4.80	0.21		4.85	0.16	
oac-17	4.48	0.23		5.13	0.18	
clec-109	4.58	0.18		5.05	0.18	
F17B5.4	5.00	0.14	8.2E-03	5.40	0.15	
clec-110	4.33	0.25		5.40	0.14	
ZK1225.1	4.58	0.20		5.18	0.17	
ZK1225.2	4.68	0.24		5.40	0.12	
ZK1225.4	3.55	0.16	2.7E-04	5.05	0.21	
ZK1225.5	4.00	0.17		4.93	0.18	3.5E-02
ssp-31	4.48	0.16		5.25	0.12	
ZK1053.3	4.05	0.17		5.03	0.20	
F44F1.1	5.33	0.11	1.8E-03	5.18	0.18	
F44F1.3	4.80	0.17		na	na	
F44F1.4	5.20	0.14	6.1E-04	5.63	0.12	
F44F1.6	3.65	0.15	9.3E-04	4.55	0.18	5.4E-04
vet-6	4.28	0.18		5.33	0.12	
sepa-1	4.95	0.18		5.48	0.08	5.0E-02
T04D3.1	4.63	0.18		4.80	0.16	
gcy-35	4.38	0.17		5.15	0.19	
T04D3.5	3.95	0.20		5.00	0.20	
W08E3.4	4.63	0.18		5.28	0.18	
Y40B1A.1	3.98	0.20	2.2E-02	4.58	0.16	1.6E-02
C01A2.3	4.43	0.20		na	na	
C01A2.6	4.63	0.14		na	na	
W05H12.2	4.43	0.21		4.75	0.15	
fbxb-66	4.33	0.24		4.68	0.20	
eif-3.J	4.13	0.21		4.28	0.14	1.3E-04
agef-1	3.98	0.22	2.8E-02	na	na	
W09C5.7	4.63	0.18		5.25	0.17	
W04A4.3	4.33	0.19		5.43	0.16	
W04A4.5	4.30	0.18		4.78	0.18	
Y6B3B.1	4.40	0.23		5.30	0.16	

Gene Name	RNAi in:					
	let-60(gf)	SE[1]	p[2]	*ewIR17; let-60(gf)*	SE[1]	p[2]
Y6B3B.3	4.48	0.16		4.83	0.21	
Y6B3B.4	3.53	0.14	8.4E-05	4.78	0.20	1.2E-02
Y6B3B.5	4.25	0.18		4.45	0.18	
Y6B3B.9	na	na		3.73	0.17	1.5E-09
Y37H9A.2	3.88	0.16	1.7E-02	5.05	0.17	
Y37H9A.3	4.08	0.20		5.13	0.16	
W04A8.1	4.13	0.17		5.38	0.12	
W04A8.2	4.35	0.24		4.95	0.19	
W04A8.3	4.43	0.18		5.63	0.11	
W04A8.5	4.53	0.14		5.13	0.13	
W04A8.6	3.93	0.15	2.4E-02	4.95	0.18	4.7E-02
ins-30	4.43	0.20		5.33	0.18	
fbxb-101	4.53	0.21		5.50	0.15	
tfg-1	3.83	0.17	1.2E-02	4.08	0.22	9.1E-06
kal-1	4.35	0.20		4.90	0.21	4.7E-02
ZK849.1	4.53	0.20		5.23	0.19	
ZK849.5	4.93	0.16		5.40	0.12	
empty vector	4.55	0.07		5.16	0.06	
